# *Bifidobacterium longum* 1714 as a translational psychobiotic: modulation of stress, electrophysiology and neurocognition in healthy volunteers

**DOI:** 10.1038/tp.2016.191

**Published:** 2016-11-01

**Authors:** A P Allen, W Hutch, Y E Borre, P J Kennedy, A Temko, G Boylan, E Murphy, J F Cryan, T G Dinan, G Clarke

**Affiliations:** 1 APC Microbiome Institute, Biosciences Building, University College Cork, Cork, Ireland; 2 Department of Psychiatry and Neurobehavioural Science, Biosciences Building, University College Cork, Cork, Ireland; 3 INFANT Research Centre, University College Cork, Cork, Ireland; 4 Department of Pediatrics and Child Health, University College Cork, Cork, Ireland; 5 Department of Electrical and Electronic Engineering, University College Cork, Cork, Ireland; 6 Alimentary Health Ltd., Cork Airport Business Park, Cork, Ireland; 7 Department of Anatomy and Neuroscience, Western Gateway Building, University College Cork, Cork, Ireland; 8 Department of Anatomy and Neuroscience, Biosciences Building, University College Cork, Cork, Ireland

## Abstract

The emerging concept of psychobiotics—live microorganisms with a potential mental health benefit—represents a novel approach for the management of stress-related conditions. The majority of studies have focused on animal models. Recent preclinical studies have identified the *B. longum* 1714 strain as a putative psychobiotic with an impact on stress-related behaviors, physiology and cognitive performance. Whether such preclinical effects could be translated to healthy human volunteers remains unknown. We tested whether psychobiotic consumption could affect the stress response, cognition and brain activity patterns. In a within-participants design, healthy volunteers (*N=*22) completed cognitive assessments, resting electroencephalography and were exposed to a socially evaluated cold pressor test at baseline, post-placebo and post-psychobiotic. Increases in cortisol output and subjective anxiety in response to the socially evaluated cold pressor test were attenuated. Furthermore, daily reported stress was reduced by psychobiotic consumption. We also observed subtle improvements in hippocampus-dependent visuospatial memory performance, as well as enhanced frontal midline electroencephalographic mobility following psychobiotic consumption. These subtle but clear benefits are in line with the predicted impact from preclinical screening platforms. Our results indicate that consumption of *B. longum* 1714 is associated with reduced stress and improved memory. Further studies are warranted to evaluate the benefits of this putative psychobiotic in relevant stress-related conditions and to unravel the mechanisms underlying such effects.

## Introduction

Recent years have seen growing research interest in the possibility of targeting the gut microbiome to beneficially impact on brain and behavior. A promising strategy in this field is that of psychobiotics—live microorganisms that convey a benefit upon the host's mental health when consumed in adequate quantities.^1^ There is increasing interest in the impact of putative psychobiotics upon central nervous system processes, especially stress, mood, anxiety and cognition.^2, 3^ Preclinical research has indicated that chronic probiotic administration can reduce anxiety-like and depressive-like behavior, and can normalize associated physiological outputs such as corticosterone, noradrenaline, brain-derived neurotrophic factor and immune function.^4, 5, 6, 7^

Although most of the evidence in this area comes from animal studies, specific probiotic strains have shown potential for symptom alleviation in irritable bowel syndrome (IBS),^8, 9^ a stress-related brain–gut axis disorder associated with high rates of psychopathology^10^ as well as altered hypothalamic–pituitary–adrenal axis activity^11^ and cognition.^12, 13^ A number of encouraging proof-of-principle studies in healthy human volunteers have now demonstrated that multistrain probiotics, fermented drinks containing probiotics or prebiotics can alter resting brain activity, cognitive performance, baseline physiological stress outputs and self-reported psychological variables.^5, 14, 15, 16, 17, 18^ It is, however, unclear whether the use of single putative psychobiotic strains is also a viable approach in humans and whether a discovery pipeline can be developed to inform superior candidate strain selection as well as evaluation across a broader range of relevant parameters.

We have recently proposed a precision strategy in the use of psychobiotics for targeting stress-related central nervous system disorders including anxiety and depression.^1^ The rational selection of candidate strains identified in well-validated preclinical screening platforms is a more logical approach to guide the challenging move toward clinically useful psychobiotics. Accordingly, in preclinical studies, we have identified the *Bifidobacterium longum* 1714 strain, which selectively improves stress-related behaviours, physiology and cognitive performance.^19, 20^ The current study investigated whether these preclinical effects could be translated to healthy human volunteers.

We examined the effects of the 1714 strain compared with placebo on daily reported stress and the psychobiological response to an acute, controlled stressor.^21, 22^ We also assessed cognitive performance; in addition to our previous preclinical findings indicating improved memory, there is existing evidence that consumption of probiotics can affect performance on sustained attention performance,^18^ as well as in brain activity during social cognition.^15^ We thus assessed cognitive performance on tests assessing memory, sustained attention, social cognition and emotional processing. We also assessed brain activity in frontal, parietal and central regions using electroencephalography (EEG) following 4-week supplementation with the 1714 strain in comparison with placebo, as these regions have been associated with memory and sustained attention^23, 24^ and are sensitive to anxiolytics.^25^

## Materials and methods

The research described received approval from the Clinical Research Ethics Committee of the Cork Teaching Hospitals (Protocol Number: APC044). Informed consent was obtained from all participants, who were free to withdraw from the study at any time.

### Design

A repeated measures, placebo-controlled design was employed. A repeated measures design was employed in order to exclude the effects of individual differences across variables. Participants were screened at an initial visit for psychiatric disorder using the MINI International Neuropsychiatric Interview (MINI^26^), and demographic and baseline psychological information was collected. Following screening, participants completed neurocognitive visits and acute stress visits. Participants were then administered placebo for four weeks followed by the 1714 strain for four weeks, with the second and third set of visits following the placebo and probiotic phases. Participants also completed a 2-week post-probiotic follow-up. Participants filled in daily online questionnaires from the end of the first set of visits to the end of follow-up.

### Participants

With a power of 0.8 for a one-way analysis of variance, a minimum sample size of 20 was required to demonstrate an effect sized *f*=0.3 at *α*=0.05.^27^ The study was completed by 22 healthy male volunteers (see Table 1 for detailed participant characteristics). Male participants were selected to avoid the need to control for menstrual cycle, which can impact upon cortisol output and other readouts. Participants were aged between 18 and 40 years of age. Exclusion criteria were as follows: having a significant acute or chronic illness; having a condition, following a diet or taking a medication that would interfere with study objectives, pose a safety risk or confound the interpretation of the study results; evidence of immunodeficiency, bleeding disorder or coagulopathy; English not being participant's first language; colour blindness, dyslexia or dyscalculia; smoking; self-report habitually taking any probiotic products; receiving any treatment involving experimental drugs (see Supplementary Figure 1 for recruitment flowchart).

### Materials

Both placebo and probiotic sticks contained maltodextrin and magnesium stearate; probiotic sticks additionally contained 1 × 10^9^ colony-forming units per stick of the 1714 strain. Participants were instructed to take one stick each morning by mixing the contents of the stick into milk and drinking.

#### Self-report

Online questionnaires were completed each day: the Cohen Perceived Stress Scale,^28^ and a question on whether participants had taken their probiotic that day. Online questionnaires were administered using limesurvey software.^29^

#### Cognitive tasks

Tests from the Cambridge Neuropsychological Test Automated Battery (CANTAB^30^) were presented on a touch-screen monitor, Sahara i440D Slate Tablet PC (Sand Dune Ventures, Tablet Kiosk, Torrance, CA, USA) running CANTABeclipse software (Cambridge, UK). The researcher provided verbal instructions to participants from a standardised script. Tests were presented in different orders for different participants, using a Latin square design, to avoid effects of fatigue for tests completed later in the session. The test battery lasted ~45 min in total. The Paired Associate Learning (PAL) test was used to assess conditional learning of pattern–location associations. Paired associate learning performance has shown sensitivity to functional changes in the hippocampus^31^ and frontal lobes.^23^ The parallel mode (which presents different shapes at each visit) was used in order to avoid practice effects.

Rapid visual information processing was used to assess sustained attention. Performance on this task activates a frontoparietal network of brain regions,^24^ and a modified version of this task is sensitive to changes in quantitative frontal EEG.^32^

Emotion Recognition Task was used to assess social cognition. Functional magnetic resonance imaging has previously demonstrated probiotic effects on a network of brain regions involved in emotional and viscerosensory processing in healthy controls.^15^

In addition to tests from the CANTAB battery, we assessed emotional processing using an Emotional Stroop.^33^ The Stroop test was presented on the same high-resolution touch-screen monitor used with the CANTAB battery. The emotional Stroop is associated with activation in the anterior cingulate cortex.^34, 35^ Positively, negatively and neutrally valenced words were presented, matched for length in letters, orthographic neighbourhood size (that is, the number of words which differ from a given word by only one letter) and frequency of use.^36^

We used an auditory oddball task to study the P300 event-related potential, which has been shown to be sensitive to anxiolytic effects at the cerebral electrode locations assessed in the current study.^25^ See Supplementary Table 1 for detailed description of the cognitive tasks.

### Neurocognitive assessment

Prior to EEG testing participants were asked to refrain from caffeine on the morning of their experimental session, as well as ensuring they got a good night's sleep, to remove any piercings and avoid wearing hair gel. All EEG measurements were made using a Neuroscan, SynAmps 2 Amplifier and Neuroscan 4.3.1 acquisition software (Compumedics, Abbotsford, VIC, Australia). EEG data were recorded at a sampling rate of 1000 Hz. Scalp electrodes were attached at midline positions Fz, Pz and Cz, according to the international 10/20 system, as well as mastoid electrodes and a reference electrode on the nose. Vertical eye movements were detected using electrodes attached above and below the orbit of the left eye and horizontal eye movements were monitored by electrodes at the right and left outer canthi. Electrodes were applied to the scalp using soft paste and secured using tape and an elasticated hat.

Following a resting EEG recording, the cognitive tasks were completed (see Cognitive tasks). EEG was recorded continuously during performance of the auditory oddball task, but was not measured during the other cognitive tasks to avoid artefacts in the EEG readings.

### Resting EEG

EEG measures of absolute power in the delta (1.5–3.5 Hz), theta (4–7.5 Hz), alpha1 (8–9.5 Hz), alpha2 (10–12.5 Hz), beta1 (13–17.5 Hz) and beta2 (18–25.5 Hz) frequency bands were taken for an initial five minute period with eyes closed (based on Romano-Torres et al.^37^). Participants were requested to relax and sit still with their eyes closed while resting EEG was recorded. Resting EEG has been shown to be consistent across repeated visits,^38^ and so should not be vulnerable to carryover effects.

### Acute stress procedure

We employed the socially evaluated cold pressor test (SECPT)^21^ as a combined psychological and physiological stressor procedure, which has been shown not to induce hypothalamic–pituitary–adrenal axis habituation across repeated exposures.^39^ Participants were required to avoid alcohol for 24 h prior to the visit, as well as caffeinated beverages on the day of the stress procedure and strenuous exercise from 1400 hours the day before, and to fast for 2 h prior to testing. Pre-stress, the participant completed the state items from the state-trait anxiety inventory,^40^ and a baseline saliva sample was taken. The baseline measures were followed by a 5-min resting phase. Following the 5-min resting phase, another saliva sample was taken. The participant read the instructions for the SECPT and the experimenter answered any questions. The participant then completed the SECPT (see Supplementary Table 2 for full details). Following completion of the SECPT the participant completed the post-stress state anxiety questionnaire. Further saliva samples were taken 1 min after the cessation of the stressor, as well as 10, 20, 30, and 60 min post-stressor cessation.

### Analysis

#### EEG analysis

**Resting EEG, frequency domain** Data were examined for eyeblink artefacts. After correction for eyeblinks, data were epoched, with an epoch duration of 2 s. A linear detrend based on the entire sweep was applied to the epoched file, which was then baseline corrected for the pre-stimulus interval. A band pass filter (0.1–35 Hz, IIR, 24 dB/octave) was applied. Following a further visual inspection for artefacts and baseline correction the epochs were averaged. Multiplication of data by a 25% cosine window was applied, as this has been used previously in examining anxiolytic effects.^41^ EEG measures of absolute power were extracted in the delta (1.5–3.5 Hz), theta (4–7.5 Hz), alpha1 (8–9.5 Hz), alpha2 (10–12.5 Hz), beta1 (13–17.5 Hz) and beta2 (18–25.5 Hz) frequency bands.

**Resting EEG, time domain** The EEG signal was downsampled from 1000 Hz to 256 Hz with an antialiasing filter set at 128 Hz. The filtered EEG signal was segmented into 1 s window without overlap. Curve length, number of maxima and minima, root mean squared amplitude, Hjorth parameters ^42^ (activity, mobility and complexity), zero crossings (raw epoch, Δ, ΔΔ), autoregressive modelling error (model order 1–9), nonlinear energy, variance (Δ, ΔΔ) were calculated using MATLAB. Mobility is an estimate of root-mean-square frequency. Mobility=std(*y*(*t*)d*y*/d*t*)/(*y*(*t*)), where the *y*(*t*) is the EEG amplitude at time, *t*.

**Event-related potential** Data were divided into epochs of 300 ms duration (200 to 500 ms post-oddball stimulus). Following a linear detrend and a baseline correction, data were visually examined for any artefacts and these were removed. Oddball epochs were averaged, and a band pass filter and further baseline correction were applied. The peak frequency was identified between 200 ms and 500 ms post-oddball for the P300.

### Sample analysis

Salivary cortisol was analysed using Enzo Life Sciences (Exeter, UK) enzyme-linked immunosorbent assay (ELISA) kits (Catalogue no: ADI-901-071) according to manufacturer's instructions. Lower limit of detection=0.16 nmol l^−1^. Inter and intra-assay coefficients of variability were 11.24% and 8.2%, respectively.

### Statistical analysis

Data were analysed using SPSS 21 (IBM, Armonk, NY, USA). Repeated measures analysis of variance and *t*-tests (two-sided) were used to examine differences between conditions, and non-parametric equivalents (Friedman and Wilcoxon, respectively) were used where parametric assumptions were violated. Areas under the curve with respect to ground (AUCg) were also calculated,^43^ and analysed in the same manner. EEG data for three participants were removed (two had excessive levels of artefacts and one participant suffered a tension headache when the EEG equipment was attached). Daily questionnaire data for three participants were removed due to lack of response to online questionnaires.

## Results

### Acute stress response to socially evaluated cold pressor test

**Figure 1:**
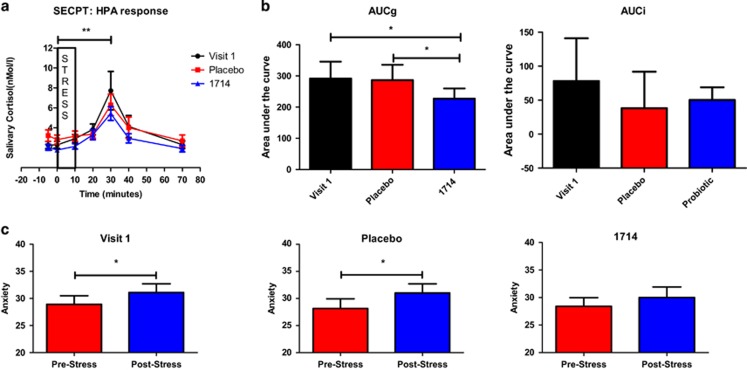
The socially evaluated cold pressor test (SECPT) elevated cortisol, and cortisol output and the increase in anxiety were reduced post-1714. (**a**) Salivary cortisol during socially evaluated cold pressor for each visit. (**b**) Total cortisol output for each visit, as measured by area under the curve with respect to ground (AUCg) and area under the curve with respect to increase (AUCi). (**c**) State anxiety (STAI) pre- and post-stressor at each visit. Lower scores indicate lower subjective anxiety. Error bars represent the s.e.m. *P<0.05, **P<0.001. HPA, hypothalamic-pituitary-adrenal.

The stressor significantly increased salivary cortisol at all visits. Salivary cortisol was significant elevated at its peak (30 min following onset of stress) compared with immediately pre-stress (time 0) for all three conditions (visit one: *T*=5, *P*<0.001, *r*=0.8, post-placebo: *T*=4.67, *P*<0.001, *r*=0.78, post-1714: *T*=2, *P*<0.001, *r=*0.94), but there was not an interaction between probiotic condition and stressor time point, F(1.86, 37.11)=1.81, *P*=0.18 (Greenhouse-Geisser adjusted; Figure 1a). Nonetheless, total cortisol output, as measured by AUCg, was significantly affected by probiotic condition, *χ*^2^(2)=8.67, *P*<0.05, although area under the curve with respect to increase (AUCi) was not affected, *χ*^2^(2)=3.71, *P*>0.05 (Figure 1b). For AUCg, cortisol output was lower post-1714, both compared with the post-placebo, *T*=10.69, *P*=0.05, *r*=0.42, and compared with the first visit, *T*=10.94, *P*<0.05, *r*=0.45.

There was a main effect of stressor on reported state anxiety, F(1, 21)=6.39, *P*=0.02. State anxiety was significantly elevated post-stressor at both visit one, *T*=8.58, *P*<0.05, *r*=0.43, and post-placebo, *T*=7.7, *P*<0.01, *r*=0.57. However, this was not the case post-1714, *T*=9.13, *P*>0.05, *r*=0.12 (Figure 1c). The mean time participants kept their hand in the water was *M*=157.5 s, s.d.=47.3 for visit 1, *M*=167.7, s.d.=39.5 post-placebo and *M*=170.2, s.d.=38 post-1714.

### Reported daily stress and bowel satisfaction

**Figure 2:**
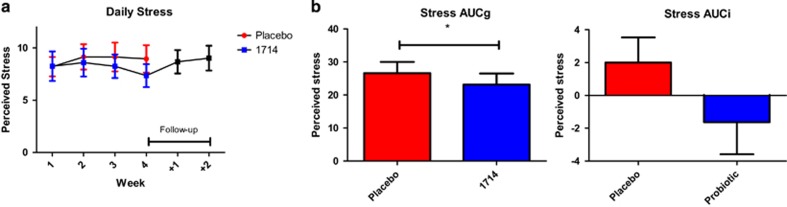
The probiotic condition was associated with reduced daily stress. (**a**) Daily stress levels during placebo phase, 1714 phase, and follow-up period. (**b**) Overall stress, as measured with AUCg and AUCi, during placebo phase, 1714 phase. *P<0.05. AUCg, area under the curve with respect to ground; AUCi, area under the curve with respect to increase.

Daily stress levels were broadly similar in both conditions during their first week, but became marginally lower in the 1714 condition by week 4, *t*(18)=1.95, *P*=0.07, Cohen's *d*=0.44. Further, stress levels returned to a higher level during the 2-week follow-up period (Figure 2a). Overall stress, as measured with AUCg, was significantly lower during the 1714 condition compared with the placebo, *t*(18)=2.32, *P*=0.03, Cohen's *d*=0.53, and AUCi was lower in the 1714 condition, although this was not statistically significant, *t*(18)=1.44, *P*>0.05, Cohen's *d*=0.33 (Figure 2b). There was reduced AUCg and AUCi for bowel satisfaction in the 1714 condition (Supplementary Figures 2A and B), due to a brief, non-significant reduction in reported bowel habit satisfaction at week 2 of the 1714 condition, but this did not persist (Supplementary Figure 2C).

### Neurocognitive performance

**Figure 3:**
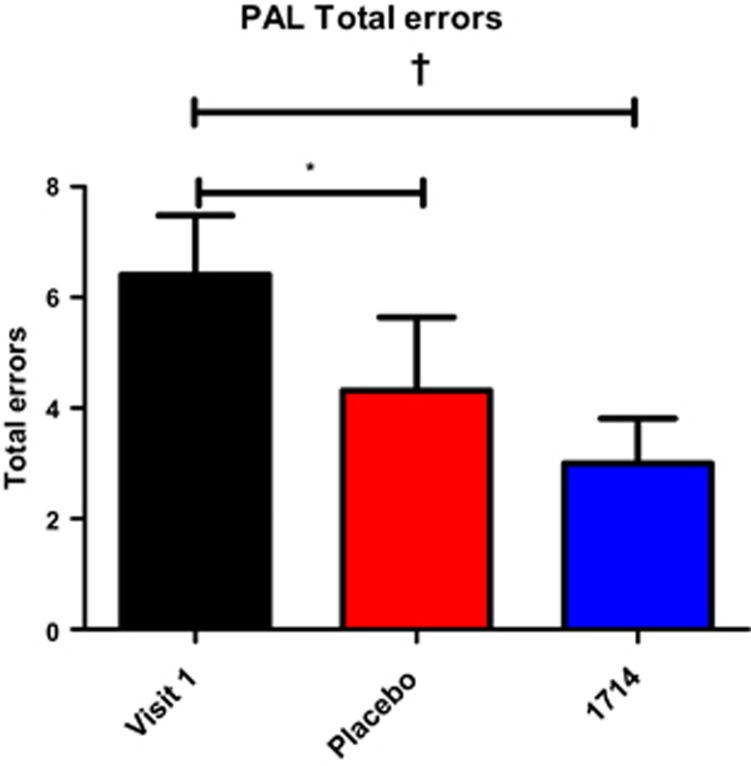
Total errors on the paired associate learning task. *P<0.05; ^†^*P*<0.01. PAL, Paired Associate Learning test.

Total errors on the paired associate learning task were significantly affected by condition, *χ*^2^(2)=10.46, *P*<0.01. Compared with the first visit, participants made fewer errors on the paired associate learning test both at post-placebo, (*T*=7.5, *P*<0.05, *r*=0.5) and post-1714, (T=6.63, *P*<0.01, *r*=0.59), a subtle effect at post-1714 that was greater than the placebo response (Figure 3). See Supplementary Table 3 for full details of results.

### EEG

**Figure 4:**
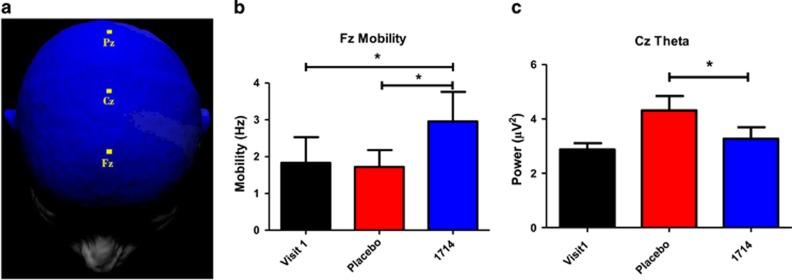
Post-1714, Fz mobility was increased and Cz theta power was decreased. (**a**) Electrode position of Fz, Cz and Pz. (**b**) EEG mobility at Fz. (**c**) Theta power at Cz. *P<0.05. EEG, electroencephalogram.

Fz mobility differed significantly across conditions, *χ*^2^(2)=13.37, *P*=0.01 (see Figures 4a for electrode positions). Mobility was significantly higher post-1714 compared with both post-probiotic, *T*=37, *P*=0.02, *r*=0.31 and visit one, *T*=39, *P*=0.02, *r*=0.3 (Figure 4b). Cz theta power differed significantly across 1714 conditions, *χ*^2^(2)=10.31, *P*<0.01. Cz theta power was significantly lower post-1714 compared with post-placebo, *T*=8, *P*<0.05, *r*=0.57, although theta power at post-1714 did not differ from visit one (Figure 4c). There was no impact of 1714 on P300 latency or amplitude (Supplementary Figure 3).

## Discussion

The current research indicates that a putative psychobiotic, the *B.* *longum* 1714 strain, which has shown anti-stress and procognitive effects in healthy mice^19, 20^ can also ameliorate both the physiological and psychological response to an acute stressor, as well as longer-term daily self-reported psychological stress, in healthy human adults. Furthermore, there was a subtle improvement over placebo in visuospatial memory performance post-psychobiotic, as well as an EEG profile consistent with improved memory. Whereas previous studies in healthy humans have often examined probiotic cocktails, we believe this is the first study to examine the effect of a single psychobiotic strain upon a wide range of stress outcomes and neurocognitive evaluations informed by a preclinical screening platform.^19, 20^ We employed a repeated measures design to control for potential effects of individual differences, with carefully selected cognitive and stress challenges that are not associated with habituation over repeated testing within this timeframe.

Following the psychobiotic intervention, cortisol output during the SECPT was lower, and the increase in self-reported anxiety was no longer significant. Interestingly, in our prior animal studies we also showed there was a blunted physiological response to stress, albeit manifested as a reduction in stress-induced hyperthermia, without any alteration in corticosterone output.^19^ This difference highlights the challenges in translating the findings from animal studies to human populations and reinforces the need for multiple stress and behavioural readouts in preclinical screening platforms. The psychobiotic effect on the acute stress response is complemented by a reduction in daily perceived stress that is consistent with previous findings that a probiotic intervention can affect subjective everyday stress.^5^

The subtle improvement in visuospatial memory following probiotic administration is consistent with preclinical findings of enhanced learning and memory in response to this strain.^20^ This cognitive effect was further supported by the observation of enhanced frontal midline (Fz) mobility using EEG. Activity at Fz is representative of prefrontal cortex activity,^44^ and the prefrontal cortex is associated with paired associate learning.^23^ As prefrontal activity appears to be specifically associated with top–down processing during associative memory,^23^ the current EEG findings thus offer a potential insight into the timing of cognitive processes underlying the observed changes in paired associate performance, by suggesting that the impact of the psychobiotic on cognitive performance may be due to an effect on top–down processing, rather than simply affecting retention. Mobility has previously been shown to be higher in healthy controls compared with patients with Alzheimer's disease,^45^ a neurodegenerative disorder with pronounced and progressive memory deficits.^46^ Although Alzheimer's disease is a severe clinical condition, it is worth noting that the observed effects in Alzheimer's patients were stronger frontocentrally, which is where the change in mobility was observed post-psychobiotic. The reduction in theta power post-psychobiotic compared with post-placebo is also consistent with improved memory performance.^47^

Further research is warranted to further elucidate mechanisms by which the 1714 strain could impact upon stress and neurocognition. It is possible that the reduction in daily stress and acute stress response may account for the observed effect on visuospatial memory. PAL test performance is dependent upon the hippocampus,^31, 48^ which has a high proportion of glucocorticoid receptors,^49^ and PAL test performance is poorer in subjects with stress-related disorders with associated hypothalamic–pituitary–adrenal axis dysfunction, such as IBS.^13^ *B. longum* NCC3001 enhanced hippocampal brain-derived neurotrophic factor messenger RNA and reduced anxiety-like behavior in mice infected with the non-invasive parasite *T Muris*^50^ and *B. breve* 6330 increased brain-derived neurotrophic factor total variants in healthy rats.^51^ It is thus of interest whether brain-derived neurotrophic factor levels and neurogenesis are heightened in humans following psychobiotic ingestion, thus having a key role in the impact of psychobiotic consumption upon the central nervous system. Furthermore, given previous evidence that psychobiotic effects in rodents were dependent upon the vagus nerve,^7^ it is possible that vagal activity may mediate such psychobiotic effects. However, strain-specific mechanisms are likely and need to be investigated on a case by case basis.

These results provide the impetus for further evaluation of this psychobiotic in pathological conditions. This could initially target stress-related brain–gut axis disorders with a cognitive component such as IBS, which has been found to be associated with a deficit in visuospatial memory performance, evident in PAL test performance,^12, 13^ and we have shown PAL performance to be subtly improved by the 1714 strain. Furthermore, the observed psychological effects of psychobiotics warrant research in other stress-related disorders including depression and anxiety.

In line with the predicted impact from preclinical research in this specific strain, the 1714 strain is a promising candidate psychobiotic strain associated with attenuated responses to psychological and physiological stress and a modest improvement over placebo in cognitive performance, as well as with altered resting EEG output in healthy volunteers. Further studies are warranted to evaluate the benefits of this putative psychobiotic in relevant stress-related conditions and to unravel the mechanisms underlying such effects.

## Figures and Tables

**Table 1:** Participant characteristics

Age, years	*M*=25.5 (1.2)
BMI	*M*=24.8 (0.7)
Alcohol consumption (units per week)	*M*=7.5 (1.3)
Years of Education	*M*=18.6 (0.6)
Trait anxiety (STAI)	*M*=29.9 (1.7)
Depression (BDI)	*M*=3.6 (0.9)
Stress (CSS)	*M*=9 (1)
IQ (NART estimated)	*M*=107.5 (1.17)
Occupation	Doctor, 3
	Psychologist, 1
	Fire service, 1
	Sculptor, 1
	Medical student, 5
	Postgraduate student, 2
	Student (other), 9

Abbreviations: BDI, Beck Depression Inventory^52^; BMI, body mass index; CSS, Cohen Perceived Stress Scale^28^; IQ, intelligent quotient; *M*, mean; NART, National Adult Reading Test^53^; STAI, State-Trait Anxiety Inventory^40^. S.e.m. in parentheses.
