# Supplementary material for: Bifidobacterium longum 1714 as a translational psychobiotic: modulation of stress, electrophysiology and neurocognition in healthy volunteers
Source: Transl Psychiatry. 2016 Nov 1;6(11):e939–. doi: 10.1038/tp.2016.191 (PMC5314114; doi:10.1038/tp.2016.191)
Supplement: Supplementary Information [file tp2016191x1.doc]

**Supplementary information**


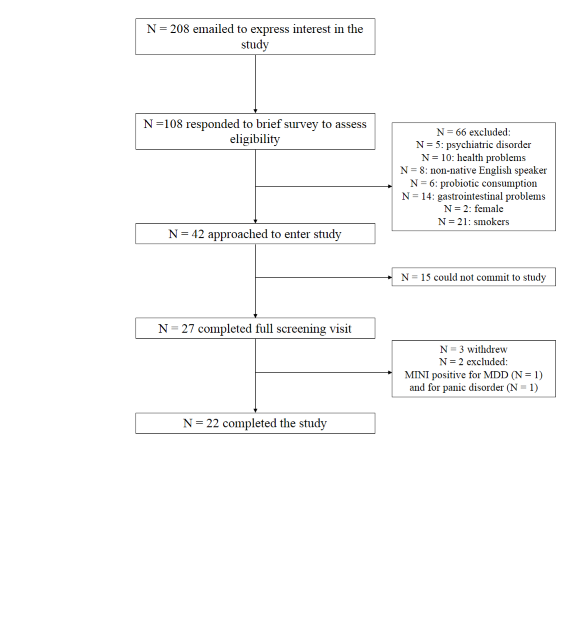


**Supplementary Figure 1:** Recruitment flowchart

**A.**

**B.**

**C.**

**Supplementary Figure 2:** Self-reported bowel satisfaction as indicated with (A). area under the curve with respect to ground (AUCg) (B). area under the curve with respect to increase (AUCi) and (C). week-per-week.


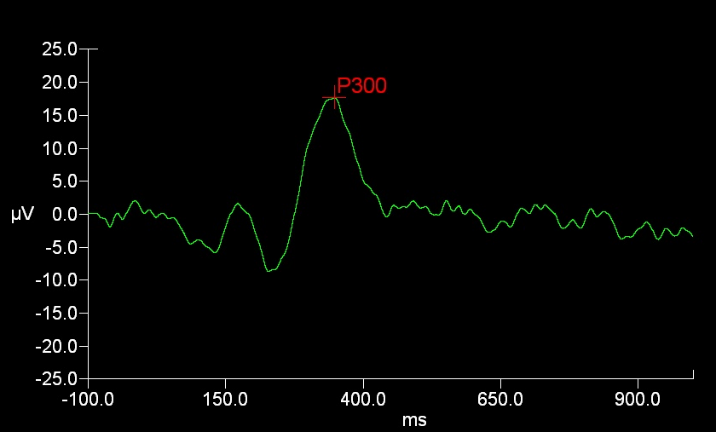


**A.**

**B.**

**C.**

**Supplementary Figure 3:** (A). Representative P300 ERP at Fz. (B). P300 latency at Fz, Cz, and Pz. (C). P300 amplitude at Fz, Cz, and Pz.

**Supplementary Table 1: CANTAB task employed**

| **Name** | **Description** | **Outcomes assessed** |
| --- | --- | --- |
| **Paired associates learning (PAL)** | The PAL assesses conditional learning of pattern-location associations. White boxes are presented on-screen and each opens in a randomized order; in some boxes a pattern is shown. Participants must remember patterns associated with different locations. In the practice phase 2 patterns are presented and after this presentation the participant must touch the boxes the patterns appeared in. Participants then have to remember 2 patterns for 1 trial, 3 for 1 trial, 6 for 1 trial and 8 for 1 trial. If the participant makes an error the patterns are re-presented to remind the participant of their locations. When all pattern locations are correctly identified the test proceeds to the next stage. If participants do not correctly identify the pattern locations within 10 trials the test terminates. The administration time was approximately ten minutes. | PAL total errors per stage (6 or 8 shapes)  mean trials required to locate all patterns correctly  first trial memory score |
| ***Rapid visual information processing (RVP)*** | The RVP test assesses sustained attention. Subjects are presented with digits appearing on screen one at a time. Participants are required to press the button on the press pad as soon as they detect target sequences of digits (e.g. 3-5-7). The test consists of a practice phase followed by a four-minute testing phase. The administration time was approximately seven minutes. | Targets correctly detected  False alarms  Reaction time |
| ***Emotion Recognition Task*** | The emotion recognition task assesses visual appraisal of emotion in the facial expressions of others. The participant is briefly shown faces displaying distinct emotions, for example happiness or anger. The participant is then required to identify the emotion as quickly as possible from a list of possible options. The administration time was approximately ten minutes. | Total correct  Total errors  Mean speed of response |
| ***Emotional Stroop*** | The Stroop test was presented on the same high-resolution touch screen monitor used with the CANTAB battery. Positively, negatively and neutrally valenced words were presented, matched for length in letters, orthographic neighbourhood size (i.e. the number of words which differ from a given word by only one letter) and frequency of use. Participants were asked to name the colour the word was printed in. Administration time was approximately ten minutes. | Total correct  Mean speed of response  Total errors |
| ***Auditory oddball task*** | The task required participants to listen to a sequence of tones. Some of these tones were frequently occurring (1,000Hz, 60 decibels; 75% of trials) and some occurred rarely (2,000Hz; 60 decibels, 25% of trials). Participants responded via a button-press to categorise each stimulus according to whether it was low-pitched or high pitched. Following a practice trial of 20 stimuli, participants completed the test phase of 200 auditory stimuli. The P300 evoked potential was measured in response to the rarely-occurring stimuli (“auditory oddball”) using a Neuroscan®, SynAmps 2 Amplifier and Neuroscan 4.3.1 acquisition software. The administration time was approximately five minutes. | P300 latency  P300 amplitude |

Supplementary Table 2: Socially evaluated cold pressor procedure

| **Pre-stress assessment (Room A)** | The participant completed the state items from the state-trait anxiety inventory, and a baseline saliva sample was taken. The baseline measures were followed by a 5-minute resting phase. Following the 5–minute resting phase, another saliva sample was taken. The participant read the instructions for the SECPT and the experimenter answered any questions that the participant had. |
| --- | --- |
| **Stress exposure**  **(Room B)** | The participant was then lead into room B, which was laid out as follows. A confederate, who was dressed in a white lab coat and maintained a neutral demeanour throughout, was seated at a table with a container of ice water (at 0-4 degrees Celsius), as well as a camera which was directed towards the participant’s face during completion of the SECPT. Paper towels were kept beside the container. The experimenter said: “*Researcher, this is subject number xxx. Subject xxx, remember that your task is to keep your hand submerged in cold water, that your facial expressions will be video recorded for later analysis, and that the researcher is specially trained to monitor non-verbal behaviour. Do you have any questions?*”  After answering any questions the experimenter left. The confederate instructed the participant to complete the cold pressor test and started the timer; during the entire procedure the confederate maintained a neutral and unencouraging demeanour. The participant was required to keep their hand in the water for up to three minutes, or for as long as they could stand. After withdrawing their hand from the water, the participant returned to room A. |
| **Post-stress assessment (Room A)** | Following completion of the cold pressor the participant completed the post-stress state anxiety questionnaire. Further saliva samples were taken 1 minute after the cessation of the stressor, as well as 10, 20, 30, and 60 minutes post-stressor cessation. |

**Supplementary Table 3: Cognitive performance following placebo and probiotic (standard errors in parentheses)**

| **Test** | **Outcome** | **Visit 1** | **Placebo** | **1714** |
| --- | --- | --- | --- | --- |
| Paired associate learning | Total errors | 6.41 (1.06) | 4.32 (1.32) | 3.0 (0.81) |
|  | Total errors (6 shape) | 0.64 (0.31) | 1.14 (0.32) | 0.73 (0.34) |
|  | Total errors (8 shape) | 5.59 (0.83) | 3.09 (1.14) | 2.27 (0.64) |
|  | First trial memory score | 17.14 (0.48) | 18.0 (0.5) | 18.31 (0.92) |
|  | Mean trials to success | 1.48 (0.08) | 1.35 (.08) | 1.25 (0.05) |
| Rapid visual information processing | Total hits | 22.32 (0.8) | 24.23 (0.71) | 24.41 (0.58) |
|  | Total false alarms | 1.18 (0.33) | 1.09 (0.25) | 0.73 (0.18) |
|  | Mean reaction time (ms) | 361.28 (10.23) | 340.84 (9.09) | 341.02 (7.61) |
| Emotion recognition test | Total correct (happy) | 26.45 (0.58) | 25.95 (0.76) | 27.23 (0.55) |
|  | Total latency (happy) | 868.44 (53.1) | 817.4 (50.4) | 759.77 (46.78) |
|  | Total correct (sad) | 24.09 (0.81) | 25.09 (0.7) | 25.0 (0.65) |
|  | Total latency (sad) | 1234.78 (98.24) | 964.07 (50.82) | 962.28 (61.67) |
|  | Total correct (angry) | 18.45 (0.73) | 19.05 (0.6) | 19.18 (0.66) |
|  | Total latency (angry) | 1326.49 (94.98) | 1105.4 (64.74) | 1030.79 (57.27) |
|  | Total correct (disgust) | 20.36 (0.93) | 21.27 (0.68) | 21.09 (0.94) |
|  | Total latency (disgust) | 1292.98 (91.35) | 1105.96 (50.9) | 1043.77 (58.0) |
|  | Total correct (fear) | 15.91 (1.18) | 16.91 (1.13) | 17.09 (1.25) |
|  | Total latency (fear) | 1325.49 (102.53) | 1146.32 (70.94) | 1129.03 (63.51) |
|  | Total correct (surprise) | 23.55 (0.52) | 22.86 (0.57) | 23.05 (0.37) |
|  | Total latency (surprise) | 1126.07 (63.26) | 1014.55 (55.17) | 981.45 (63.65) |
| Emotional Stroop | % correct (positive) | 98.85 (0.36) | 99.43 (0.23) | 98.16 (0.6) |
|  | Mean RT (positive) | 778.77 (21.26) | 788.52 (32.92) | 770.35 (27.61) |
|  | % correct (negative) | 99.08 (0.39) | 98.85 (0.4) | 99.09 (0.31) |
|  | Mean RT (negative) | 781.14 (20.96) | 801.26 (34.03) | 763.11 (23.27) |
|  | % correct (neutral) | 98.92 (0.41) | 99.14 (0.33) | 99.51 (0.23) |
|  | Mean RT (neutral) | 783.34 (22.94) | 780.29 (32.41) | 772.86 (27.46) |
